# Amblyopia, Strabismus and Refractive Errors in Congenital Ptosis: a systematic review and meta-analysis

**DOI:** 10.1038/s41598-018-26671-3

**Published:** 2018-05-29

**Authors:** Yijie Wang, Yufeng Xu, Xi Liu, Lixia Lou, Juan Ye

**Affiliations:** Department of Ophthalmology, the Second Affiliated Hospital of Zhejiang University, College of Medicine, Hangzhou, Zhejiang, China

## Abstract

Congenital ptosis may be associated with abnormalities of visual development and function, including amblyopia, strabismus and refractive errors. However, the prevalence estimates of these abnormalities vary widely. We performed a systematic review and meta-analysis to estimate the prevalence of amblyopia, strabismus and refractive errors in congenital ptosis. Cochrane, Pubmed, Medline, Embase, and Web of Science were searched by July 2017. We used random/fixed effects models based on a proportion approach to estimate the prevalence. Heterogeneity would be considered signifcant if the p values less than 0.1 and/or I^2^ greater than 50%. Subgroup analyses, meta-regression analyses and sensitivity analyses were utilized to explore the potential sources of it. A total of 24 studies selected from 3,633 references were included. The highest prevalence was revealed for myopia with 30.2% (95%CI 3.0–69.8%), followed by 22.7% (95%CI 18.5–27.8%) for amblyopia, 22.2% (95%CI 7.8–63.1%) for astigmatism, 19.6% (95%CI 16.5–23.2%) for strabismus, 17.3% (95% CI 13.1–22.9%) for anisometropia and 4.0% for hyperopia (95%CI 1.8–7.1%). Significant heterogeneity was identified across most estimates. Our findings suggest that amblyopia, strabismus and refractive errors in congenital ptosis are present in much higher percentage. This study highlights the importance of early diagnosis and timely treatment of patients with congenital ptosis.

## Introduction

Ptosis refers to either unilateral or bilateral drooping of the upper eyelid, resulting in narrowing of the palpebral fissure and covering part of the eye^1–3^. Congenital ptosis is present at birth or in the first year of life^4^. It is relatively rare compared with other congenital oculopathy. The total prevalence of congenital ptosis in general population is 0.18–1.41%^5–7^. Despite being mostly a non-progressive disease, congenital ptosis can cause cosmetic, functional and psychosocial problems in children^8^. Although most cases of congenital ptosis represent an isolated eyelid malposition, significant ocular associations or consequences are common^9^. In addition, it can result in abnormal postures such as backward tilt of the head, chin elevation and even orthopedic problems in some severe cases^4,10,11^. Most patients with congenital ptosis need surgical correction.

Amblyopia, strabismus and refractive errors have aroused much more attention globally over the past decades. Amblyopia is defined as decreased vision due to abnormal development of visual cortex in infancy or childhood^12,13^. Strabismus, also known as squint, may interfere with normal binocular depth perception and thereby cause substantial physical disturbance^12^. Amblyopia and strabismus are common reasons of low vision and are responsible for reduced life qualities of children. Refractive errors are the leading causes of blindness around the world^14^. It has been reported that the prevalence of amblyopia, strabismus and refractive errors among patients with congenital ptosis were much higher than those among the general population. Early diagnosis and treatment of congenital ptosis will contribute to prevention and management of these ocular abnormalities.

To the best of our knowledge, the prevalence of amblyopia, strabismus and refractive errors among patients with congenital ptosis vary widely across studies and have not been systemically reviewed. Against this background, we conducted the first systematic review and meta-analysis of eligible observational studies of the prevalence of amblyopia, strabismus and refractive errors in congenital ptosis.

## Results

### Summary of included studies

**Figure 1:**
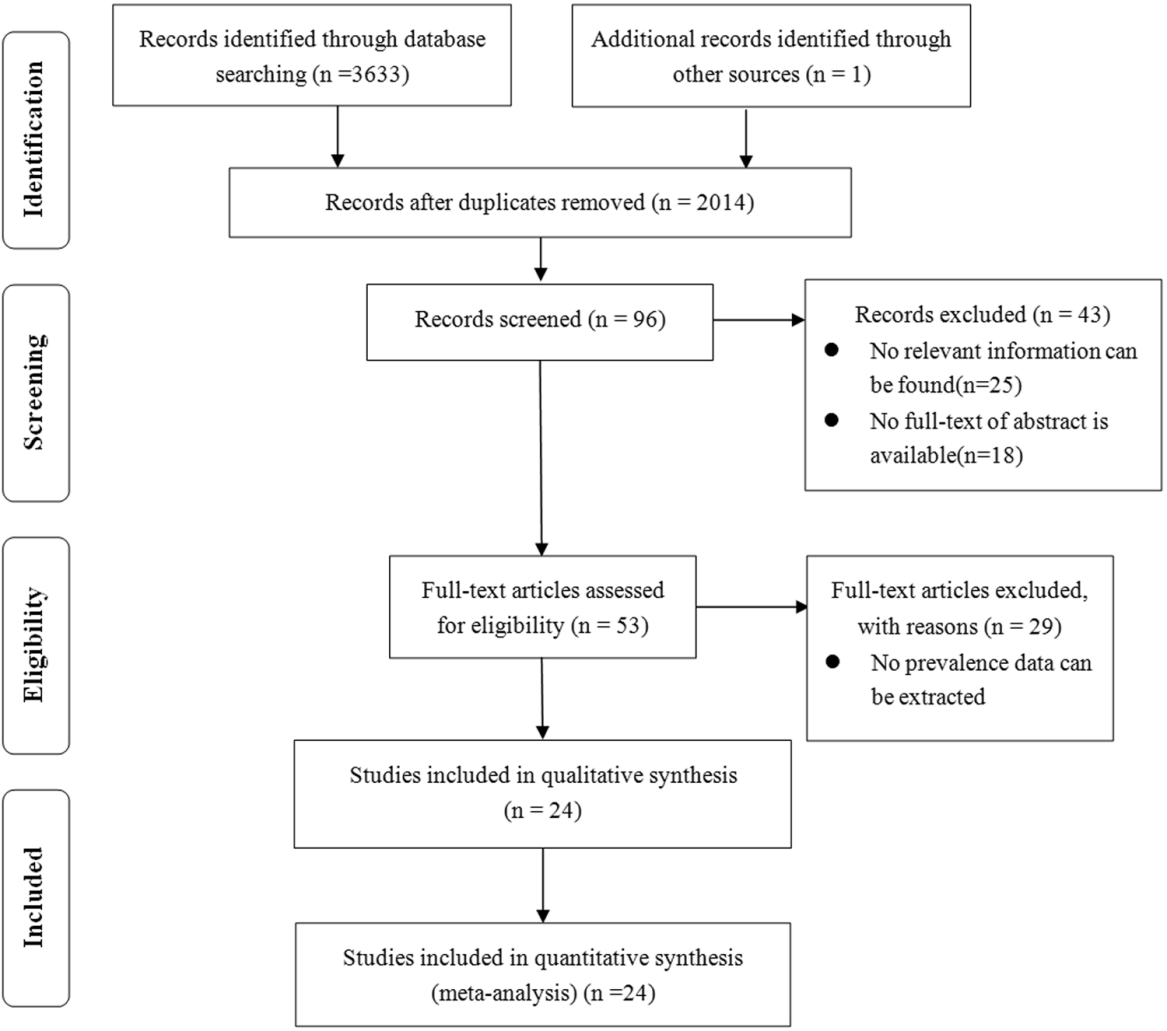
PRISMA flowchart of study selection process.

The detailed steps were given as a PRISMA flowchart (Fig. 1). A total of 3,633 articles were identified. After removal of duplicates and non-relevant studies, the abstracts of the remaining articles were reviewed. 53 articles with potentially relevant studies were further identified in full. Overall, 24 published studies were considered eligible (Table 1), including 19 studies for amblyopia^5,8,10,15–30^, 13 for strabismus^5,8,10,19,21,22,26,27,29,31–34^ and 8 for refractive errors^8,10,21,24,26,27,29,35^. Among the 24 eligible studies, 3 were population-based and 21 were hospital-based. 20 studies reviewed were written in English, 1 in Mandarin, 1 in French, 1 in Germany and 1 in Portuguese. The included studies represented four geographical regions. 10 studies were from America, 8 from Asia, 5 from Europe and 1 from Africa. The sample sizes in the included studies ranged from 36 to 216, with a combined total of 2,589. For more details, refer to Table 1.

**Table 1:** Characteristics of the included study.

Study	Disease	Region	Publication	Study Design	Setting	Mean age (year)	Male ratio	Time span (year)	sample	Quality
Anderson RL^32^	strabismus	USA	1980	cross-sectional study	hospital-based	NA	NA	3	113	8
Anderson RL^14^	amblyopia	USA	1980	cross-sectional study	hospital-based	NA	NA	3	123	8
Griepentrog GJ^15^	amblyopia	USA	2013	retrospective study	population-based	1.3	1.29	40	96	7
Griepentrog GJ^33^	strabismus	USA	2014	retrospective study	population-based	1.3	1.29	40	96	7
Berry-Brincat A^5^	amblyopia strabismus refractive errors	UK	2009	retrospective study	hospital-based	3.91	1.5	10	155 155 155	8
Dray JP^16^	amblyopia	France	2002	retrospective study	hospital-based	NA	NA	12	130	6
Gautam P^17^	amblyopia	Nepal	2016	retrospective study	hospital-based	23	NA	2	170	4
Júnior G^34^	strabismus	Brasil	2011	cross-sectional study	hospital-based	7.2	2	10	42	4
Harrad RA^18^	amblyopia strabismus	UK	1988	retrospective study	hospital-based	NA	NA	5	216 216	4
Hornblass A^19^	amblyopia	USA	1995	retrospective study	hospital-based	NA	NA	14	36	7
Ho YF^35^	strabismus	Taiwan	2017	retrospective study	hospital-based	3.86	1.8	10	319	7
Merriam WW^20^	amblyopia strabismus refractive errors	USA	1980	retrospective study	hospital-based	NA	NA	3	65 65 65	8
Skaat A^21^	amblyopia strabismus	Israel	2013	retrospective study	hospital-based	0.83	1.31	11	162 162	6
Srinagesh V^22^	amblyopia strabismus refractive errors	USA	2011	retrospective study	hospital-based	2.5	NA	4	92 87 87	7
Handor H^23^	amblyopia	Morocco	2014	retrospective study	hospital-based	10	1.75	7	44	6
Huo L^24^	amblyopia refractive errors	China	2012	retrospective study	hospital-based	16.83	NA	12	85 85	7
Abolfazl K^25^	amblyopia	Iran	2010	cross-sectional study	hospital-based	NA	1.56	2	100	7
Lin LK^26^	amblyopia strabismus refractive errors	USA	2008	retrospective study	hospital-based	NA	1.77	7	130 130 130	6
Stark N^27^	amblyopia strabismus refractive errors	German	1984	retrospective study	hospital-based	NA	NA	6	54 54 54	5
Stein A^28^	amblyopia	USA	2014	retrospective study	hospital-based	1.54	NA	18	84	7
Thapa R^29^	amblyopia strabismus refractive errors	Nepal	2010	cross-sectional study	hospital-based	16	NA	1	78 78 78	8
Hashemi H^30^	amblyopia strabismus	Iran	2015	cross-sectional study	population-based	7	1.41	1	58 58	9
Rong H^31^	amblyopia	China	2016	retrospective study	hospital-based	7.4	NA	2	187	8
Yalaz M^36^	refractive errors	Turkey	1996	retrospective study	hospital-based	15.75	1.55	5	39	4

NA, Not Available.

### Quality assessment

All of the selected articles were scored and listed in Table 1. The details could be found in Supplementary Table S1. Of all the articles included, 7 were of high quality and 17 were of moderate quality. There were no articles with low quality rating.

### Prevalence of amblyopia in congenital ptosis

**Figure 2:**
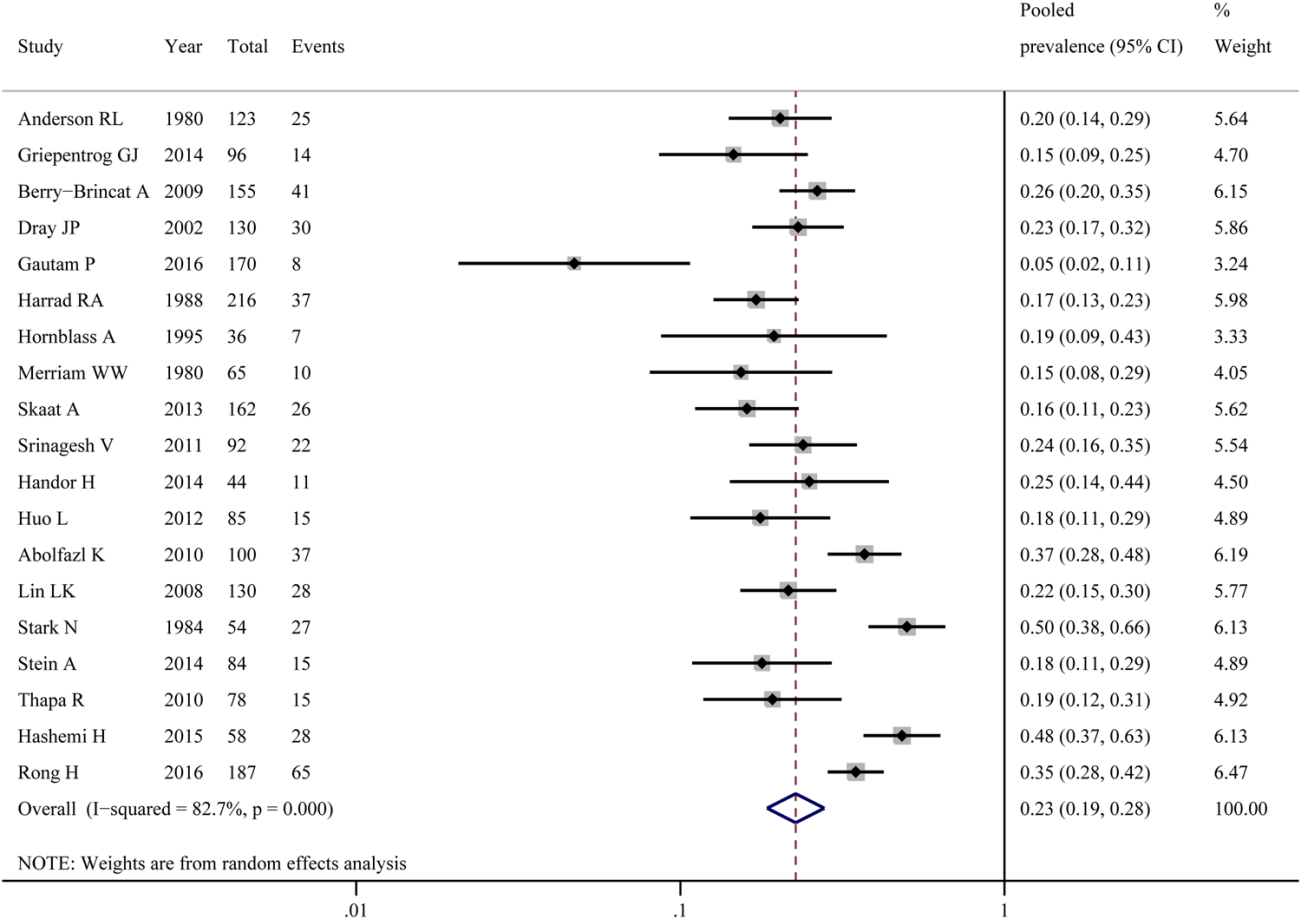
Pooled prevalence of amblyopia in congenital ptosis.

The prevalence of amblyopia extracted from each study ranged from 4.7% to 50.0% (Fig. 2); heterogeneity was substantial (χ² = 103.89, p < 0.01; I² = 82.7%). As a result, a random effects model was used. The overall pooled prevalence of 19 studies was 22.7% (95%CI 18.5–27.8%). The sensitivity analysis showed that the result was not excessively influenced by any one study. Publication bias was examined through the use of a funnel plot (Supplementary Fig. S1a), and the asymmetry was further tested by using an Egger’s test (p = 0.001) and a Begg’s test (p = 0.01). The outcomes indicated that there had a risk of publication bias, so we used the trim and fill method to recalculate the pooled prevalence (Supplementary Fig. S1b). This analysis did not change the result, suggesting that it was not affected by publication bias. The prevalence was further analyzed by subgroups (year of publication, study design, setting, sample size, time span, region and the quality of study). Although results were inconsistent, the prevalence of amblyopia in congenital ptosis did not differ substantially in all the subgroups we made (Table 2). It suggested that these grouping variables could not explain the high between-study heterogeneity in the prevalence estimates. Further analyses by meta-regression also showed that none of the factors we explored were significantly associated with heterogeneity (Table 3).

**Table 2:** Subgroup analyses for prevalence of amblyopia and strabismus in congenital ptosis.

Subgroup		Number of studies	Estimated prevalence (%; 95% CI)	I^2^ (%)
**Amblyopia**				
Year of publication	Before 2000	5	0.227 (0.133, 0.386)	88.2
	2000–2009	3	0.240 (0.201, 0.287)	0.0
	After 2009	11	0.219 (0.163, 0.295)	84.9
Region	America	7	0.198 (0.167, 0.235)	0.0
	Europe	4	0.270 (0.172, 0.424)	89.6
	Asia	7	0.228 (0.155, 0.338)	88.9
	Africa	1	0.250 (0.142, 0.440)	NA
Study design	Cross-sectional study	4	0.298 (0.195, 0.455)	84.6
	Retrospective study	15	0.210 (0.167, 0.263)	81.1
Setting	hospital-based	17	0.222 (0.181, 0.272)	80.1
	population-based	2	0.271 (0.084, 0.876)	93.6
Sample size (patients)	< 100	10	0.237 (0.170, 0.331)	82.3
	≥ 100	9	0.217 (0.167, 0.281)	83.6
Time span (years)	< 10	12	0.247 (0.188, 0.323)	86.1
	≥ 10	7	0.201 (0.168, 0.241)	24.2
Quality	4–7 (moderate quality)	13	0.208 (0.159, 0.272)	82.8
	8–11 (high quality)	6	0.270 (0.199, 0.366)	80.8
**Total**		19	0.227 (0.185, 0.278)	82.7
**Strabismus**				
Year of publication	Before 2000	4	0.240 (0.177, 0.325)	61.8
	2000–2009	2	0.151 (0.113, 0.202)	0.0
	After 2009	7	0.186 (0.150, 0.231)	41.9
Region	America	6	0.182 (0.127, 0.261)	68.3
	Europe	3	0.195 (0.140, 0.270)	56.4
	Asia	4	0.203 (0.155, 0.266)	56.0
Study design	Cross-sectional study	4	0.270 (0.209, 0.349)	28.5
	Retrospective study	9	0.177 (0.155, 0.201)	6.0
Setting	hospital-based	11	0.200 (0.165, 0.242)	62.3
	population-based	2	0.162 (0.109, 0.241)	0.0
Sample size (patients)	<100	7	0.198 (0.150, 0.261)	44.5
	≥100	6	0.193 (0.153, 0.244)	70.1
Time span (years)	<10	8	0.214 (0.167, 0.273)	62.2
	≥10	5	0.170 (0.144, 0.200)	0.0
Quality	4–7 (moderate quality)	8	0.182 (0.159, 0.208)	3.5
	8–11 (high quality)	5	0.217 (0.152, 0.310)	71.4
**Total**		13	0.196 (0.165, 0.232)	56.9

**Table 3:** Meta-regression analyses for prevalence of amblyopia and strabismus in congenital ptosis.

	Meta-regression coefficient (%)	95%CI	p
**Amblyopia**			
Year of publication, continuous	−0.002	(−0.021, 0.018)	0.839
Region (Asia vs others)	0.049	(−0.446, 0.544)	0.836
Study design	0.353	(−0.186, 0.891)	0.185
Setting	0.262	(−0.497, 1.021)	0.476
Sample size, continuous	−0.003	(−0.007, 0.002)	0.213
Time span, continuous	−0.016	(−0.043, 0.011)	0.221
Quality, continuous	0.127	(−0.041, 0.294)	0.130
**Strabismus**			
Year of publication, continuous	−0.011	(−0.023, 0.001)	0.061
Region (Asia vs others)	0.065	(−0.361, 0.491)	0.744
Study design	0.467	(0.183, 0.751)	0.004
Setting	−0.205	(−0.815, 0.404)	0.474
Sample size, continuous	−0.001	(−0.003, 0.001)	0.388
Time span, continuous	−0.012	(−0.032, 0.008)	0.231
Quality, continuous	0.004	(−0.131, 0.139)	0.947

### Prevalence of strabismus in congenital ptosis

**Figure 3:**
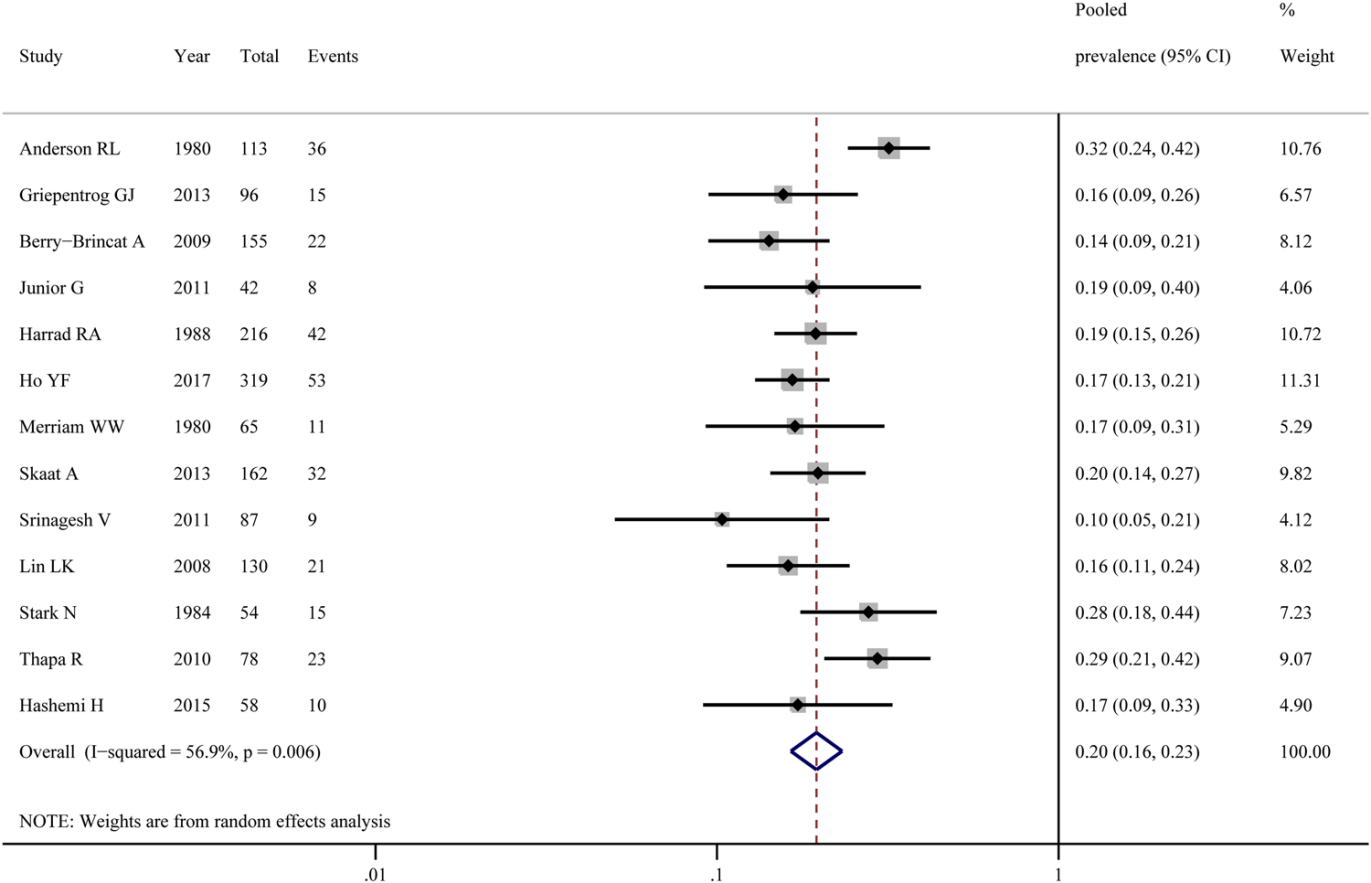
Pooled prevalence of strabismus in congenital ptosis.

Estimates of the prevalence of strabismus ranged from 10.3% to 31.9% (Fig. 3); heterogeneity was pronounced (χ² = 27.86, p < 0.01; I² = 56.9%). The random effects pooled prevalence was 19.6% (95%CI 16.5–23.2%). In the sensitivity analysis, we failed to attribute the heterogeneity to any single study. Publication bias was assessed by constructing a funnel plot (Supplementary Fig. S2) followed by an Egger’s test (p = 0.288) and a Begg’s test (p = 0.583), which indicated an insignificant level of publication bias. The results of stratified meta-analyses in subgroups were shown as below (Table 2). Prevalence for the subgroup of cross-sectional studies (27.0%, I^2^ = 28.5%) was higher than that of retrospective studies (17.7%, I^2^ = 6.0%), and there was a significant difference (95%CI 20.9–34.9% vs. 95%CI 15.5–20.1%; p < 0.01). None of other covariates were remarkable. In meta-regression analyses, we found that the study design (p = 0.004) and the year of publication (p = 0.061) were two important causes for the between-study heterogeneity (Table 3).

### Prevalence of refractive errors in congenital ptosis

**Figure 4:**
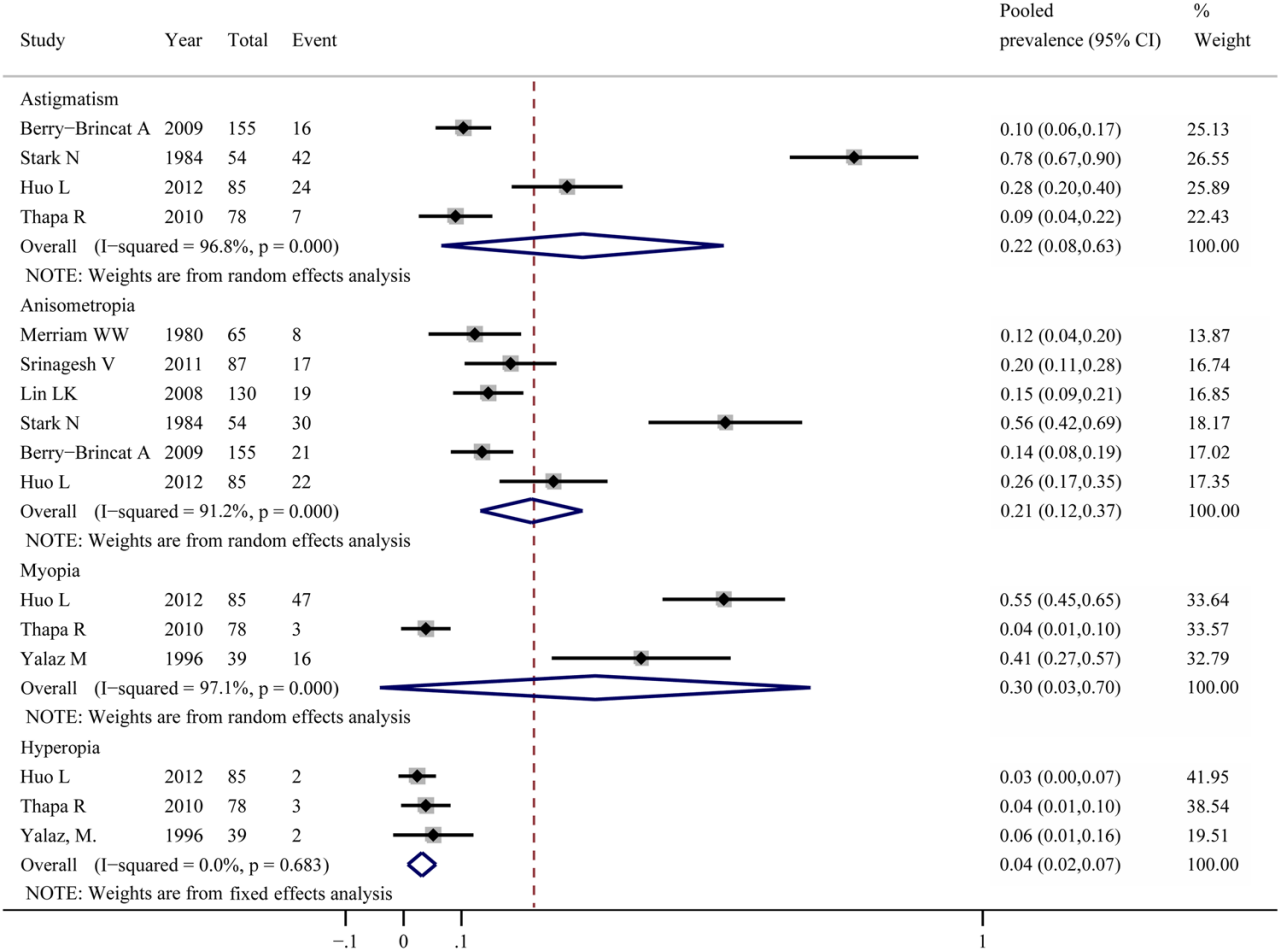
Pooled prevalence of refractive errors in congenital ptosis.

There were eight studies reporting the prevalence of refractive errors in congenital ptosis. Among them, 3 focused on the prevalence of myopia^24,29,35^, 3 on hyperopia^24,29,35^, 4 on astigmatism^8,24,27,29^ and 6 on anisometropia^8,10,21,24,26,27^. The variance-stabilising double arcsine transformation was implemented previously to calculate the prevalence of myopia and hyperopia in congenital ptosis. We did not perform funnel plots, Egger’s tests, Begg’s tests, subgroup analyses and meta-regression analyses for a lack of data in this part. The prevalence of myopia ranged from 3.8% to 55.3% (Fig. 4). There was a high level of heterogeneity (χ² = 67.87, p < 0.01; I² = 97.1%) and then a random effects model was conducted. The pooled prevalence was 30.2% (95%CI 3.0–69.8%). The prevalence of hyperopia ranged from 2.4% to 5.1% and with no evidence of heterogeneity (χ² = 0.76, p > 0.1; I² = 0.0) (Fig. 4). A fixed effects model was conducted and the overall pooled prevalence was 4.0% (95%CI 1.8–7.1%). The prevalence of astigmatism ranged from 9.0% to 77.8% (Fig. 4). The overall pooled prevalence was 22.2% (95%CI 7.8–63.1%) using a random effects model (χ² = 94.58, p < 0.01; I² = 96. 8%). Sensitivity analysis reported that no individual study affected the stability of the prevalence. The prevalence of anisometropia ranged from 12.3% to 55.6% with a pooled prevalence of 20.8% (95%CI 11.9–36.5%) using a random effects model (χ² = 57.09, p < 0.01; I² = 91.2%) (Fig. 4). Sensitivity analysis showed that the study of Stark N *et al*.^27^ substantially influenced the pooled prevalence. The prevalence was 17.3% (95%CI 13.1–22.9%) and the heterogeneity decreased to 44.5% after excluding this study.

## Discussion

This systematic review and meta-analysis of 24 studies involving 2,589 individuals demonstrated the prevalence of amblyopia, strabismus and refractive errors in congenital ptosis. The quality of all the included studies was acceptable. The highest prevalence was revealed for myopia with 30.2% (95%CI 3.0–69.8%), followed by 22.7% (95%CI 18.5–27.8%) for amblyopia, 22.2% (95%CI 7.8–63.1%) for astigmatism, 19.6% (95%CI 16.5–23.2%) for strabismus, 17.3% (95%CI 13.1–22.9%) for anisometropia, and 4.0% for hyperopia (95%CI 1.8–7.1%). It was better to consider the confidence intervals rather than the pooled results for the high heterogeneity.

Congenital ptosis is clearly related with amblyopia and always with coexistent strabismus or anisometropia^10^. Young children have often lost the best opportunity for treatment when diagnosed, which leads to a high rate of complications. The main implication of our study is that early examination and follow up of patients with congenital ptosis to monitor their visual development is necessary.

Amblyopia is one of the most common reasons for decreased vision among patients with congenital ptosis^16^. It has an estimated prevalence of 3.0% to 3.2% in the general population^15,17^. The rate among patients with congenital ptosis has been reported to be higher. In our study, the pooled prevalence of amblyopia in congenital ptosis was estimated as 22.7% (95%CI 18.5–27.8%), at an approximately 7 times greater than that in the general population. There is no definitive explanation of why the prevalence of amblyopia is higher among patients with congenital ptosis. Most authors deemed that the leading causes of amblyopia in congenital ptosis were the coexistent strabismus or significant refractive error^10,17,19,26^. Griepentrog GJ *et al*.^16^ found that eyelid occlusion of the visual axis was the main cause. Early surgery of congenital ptosis has been proved to be effective for prevention and elimination of amblyopia.

Of the 19 studies for amblyopia, a large amount of heterogeneity was identified. Sensitivity analysis showed that our results were not materially different. And there was no evidence of publication bias after applying the trim and fill method. To explore the cause of heterogeneity, subgroup analyses and meta-regression analyses were conducted. However, these analyses did not provide a clear explanation. Characteristics that we could not test might have contributed to heterogeneity, such as age, laterality, comorbidity, ptosis severity, etc.

Strabismus has an estimated prevalence of 1–5% in the general population^17^. The pooled prevalence showed that 19.6% (95%CI 16.5–23.2%) of patients with congenital ptosis suffered from strabismus, suggesting that strabismus was at least 4 times more common than that in the general population. However, it is still controversial on the precise cause for the remarkable higher prevalence of strabismus in congenital ptosis. The traditional view is that the strabismus may occur secondary to the disruption of binocularity resulting from the visual occlusion by the ptotic eyelid^31,36^. Other hypotheses center around a genetic predisposition or an intrauterine insult to the overlapping regions of the oculomotor nuclear complex or the third cranial nerve^17,37^.

Of the 13 studies for strabismus, considerable heterogeneity was found. Sensitivity analysis did not reveal significant differences. No evidence for publication bias was observed. We have found that the study design and the year of publication were two important causes for heterogeneity in our subgroup analyses and meta-regression analyses. Regarding study design, cross-sectional studies showed higher prevalence than retrospective studies did. One of the possible causes could be that some patients with strabismus were excluded for their incomplete or missed data when evaluated in retrospective studies. Further, some retrospective studies did not concern history of ptosis surgery or previous treatment for strabismus, which might result in an underestimation of the true prevalence. Older studies showed significantly higher prevalence than newer studies did. This changing trend may be due to the more attention paid to ptosis which used to be regarded as a cosmetic problem and the more effective surgical treatments used nowadays. Unknown confounding factors could still exist and thus influenced the pooled analysis.

Until now, studies on refractive errors in congenital ptosis are rare. In this study, the pooled prevalence of myopia was 30.2% (95%CI 3.0–69.8%), astigmatism 22.2% (95%CI 7.8–63.1%), anisometropia 17.3% (95%CI 13.1–22.9%) and hyperopia 4.0% (95%CI 1.8–7.1%), respectively. The proportion of different types of refractive errors in congenital ptosis varied between studies^8,38^. Gusek-Schneider *et al*.^39^ found that children with isolated congenital ptosis had more spherical and cylindrical diopters than control group. Yalaz M *et al*.^35^ proposed that in simple congenital ptosis the development of myopia and anisometropia might be due to the narrow palpebral aperture. Some studies suggested that the development of astigmatism in congenital ptosis was presumably due to eyelid tension and changes in the corneal curvature^5,25,40,41^. Ji-Sun P *et al*.^42^ recommended frequent refraction tests to ensure that the best spectacle-corrected visual acuity was obtained. And what cannot be ignored is that some refractive errors are not apparent at presentation. In this case, close follow-up is necessary, not just for refractive changes but also for other abnormalities which may progress, including latent amblyopia and strabismus.

There were 8 studies for refractive errors. Significant heterogeneity was observed in most estimates except in hyperopia. For myopia, the difference between studies might be associated with the various ethnicities and age groups of patients. It has been reported that the prevalence of myopia in East Asian countries such as China is much higher^43,44^. Huo L *et al*.^24^ deemed that long-standing congenital ptosis might produce myopia. For hyperopia, the result was robust. For astigmatism, sensitivity analysis showed that the effects of bias were not important. Kame RT *et al*.^45^ suggested that the greater tightness and narrower palpebral apertures of the Asian eyelids might lead to the greater rates of astigmatism. Besides, infants exhibited a high incidence of corneal astigmatism,and the cornea flattened with significantly reduced astigmatism as children grew older^46^. These could be potential confounding factors for the estimation. For anisometropia, sensitivity analysis showed that the study of Stark N *et al*.^27^ contributed to most of the observed heterogeneity. Limitations of this study included an imprecise definition of anisometropia and a small sample size, which might have yielded more extreme prevalence estimates.

This review has a few limitations. First, as in other meta-analyses of prevalence, substantial heterogeneity was observed^47,48^. Although we attempted to find the causes of it by conducting subgroup analyses and meta-regression analyses, the between-study heterogeneity could not be fully explained by the variables we examined. We suspect that other factors such as sex, age, laterality, comorbidity, ptosis severity, previous treatment etc. may also influence the prevalence. Therefore, the results of this meta-analysis should be prudently considered. Second, most of the available studies for our meta-analysis were hospital-based ones with limited sample sizes. Those hospitalized patients might present with more severe ptosis or other ophthalmic diseases, so the true prevalence might be higher still. Third, retrospective medical records were used in some studies. These data were taken from different ophthalmologists and the recorded information might be limited. Fourth, the definitions of amblyopia, strabismus and refractive errors were various. Actually, a detailed description of the diagnostic method was lacking in some reviewed studies. Fifth, as we did not consult unpublished articles, publication bias could not be excluded. Despite all of these limitations, it might be the first systematic review and meta-analysis to assess the prevalence of amblyopia, strabismus and refractive errors in congenital ptosis.

In conclusion, the prevalence of amblyopia, strabismus and refractive errors in congenital ptosis are much higher than those in the general population. These complications may be minimized or avoided with early surgical correction. This study highlights the importance of early assessment and timely treatment of patients with congenital ptosis. Moreover, a large-scale, multicenter-based prospective study using standard diagnostic methods and screening tools is recommended, and it will provide a more accurate estimate of the prevalence of amblyopia, strabismus and refractive errors in congenital ptosis.

## Methods

### Literature search

We preformed this systematic review and meta-analysis following the PRISMA (Preferred Reporting Items for Systematic Reviews and Meta-Analyses) statement^49^. We systematically searched all publications using Cochrane, Pubmed, Medline, Embase and Web of Science up to July 2017. We included a combination of search terms, such as Strabismus/Squint, Amblyopia/weak sight, Refractive Errors/ametropia, ptosis/blepharoptosis, etc. For the detailed search, please refer to Supplementary Appendix A. No restrictions were imposed based on language or year of publication. We also manually scrutinized the reference lists of all included studies. Titles and abstracts of studies were initially reviewed to exclude unrelated ones. Then the remaining articles were evaluated in full. All the relevant studies were independently scanned by two reviewers (YW and YX). When discrepancies occurred, consensus was achieved by consulting the senior author (JY).

### Study selection

We included studies that met the following criteria: (1) a full-text article could be obtained; (2) a cross-sectional study, case-control study, cohort study or randomized control trial; (3) the diagnosis was based on objective examination or medical records of qualified pediatricians or ophthalmologists; (4) the studies differentiated congenital ptosis from other kinds of ptosis; (5) the studies provided the extractable data of the number of patients had amblyopia, strabismus or refractive errors with congenital ptosis. Case reports, letters to the editor, drug trials, reviews or any other studies without raw or sufficient data were excluded. We chose the study with the most complete data if populations overlapped between them. All the studies were independently selected by two reviewers (YW and YX) on the basis of criteria. Disagreements between the two reviewers were resolved and adjudicated by the senior author (JY).

### Data extraction

These following characteristics were independently extracted by two reviewers (YW and XL): name of the first author, region of the study population, year of publication, study design, setting, mean age, male ratio, time span, the number of patients in the studies and the number of patients had amblyopia, strabismus or refractive errors with congenital ptosis.

### Quality assessment

The methodological quality was assessed using the checklist recommended by Agency for Healthcare Research and Quality (AHRQ)^50^. The system allowed a total score of up to 11 points. If an item was answered ‘NO’ or ‘UNCLEAR’, it was scored ‘0’; if the answer was ‘YES’, then scored ‘1’. Studies would be classified into three grades according to their scores: low quality (0–3 points), moderate quality (4–7 points) and high quality (8–11 points).

### Statistical analysis

We used the STATA (Version 12.0, Stata corporation, College Station, Texas, USA) for proportion and summary meta-analysis. Statistical tests were two-sided and used a significant threshold of p < 0.05. The prevalence of amblyopia, strabismus and refractive errors in congenital ptosis were combined to pooled prevalence respectively, and 95% confidence intervals (CIs) were calculated. They were converted into natural logarithms in advance. For data with extremely low prevalence, we handled them by the variance-stabilising double arcsine transformation previously^51^.

Heterogeneity was estimated using Cochran’s Q (reported as χ² and p values) and I^2^ statistics. P values less than 0.1 and/or I^2^ greater than 50% were considered to be high degrees of between-study heterogeneity and then a random effects model was used; otherwise, a fixed effects model was used^52^. Sensitivity analysis was performed to assess the stability of the results by sequentially omitting one study each time. Reanalyzing the remaining studies was followed. Publication bias was assessed by the funnel plot, Egger’s test and Begg’s test for sections with 10 or more studies. If publication bias was indicated, we further conducted a trim and fill analysis to evaluate the number of missing studies. Potential sources of heterogeneity were further investigated using subgroup analyses and meta-regression analyses when at least 10 studies were available. The grouping variables were as follows: year of publication (before 2000, 2000–2009 or after 2009), study design (retrospective or cross-sectional), setting (hospital-based or population-based), sample size (less or more than 100), time span (less or more than 10 years), region (different continents) and the quality of study (low, moderate or high). Meta-regression analyses were developed with the same covariates. Other grouping variables were limited by the available data.

### Data availability statement

All data are fully available without restriction.

## Electronic supplementary material

Supplementary
