# Supplementary material for: Amblyopia, Strabismus and Refractive Errors in Congenital Ptosis: a systematic review and meta-analysis
Source: Sci Rep. 2018 May 29;8:8320. doi: 10.1038/s41598-018-26671-3 (PMC5974416; doi:10.1038/s41598-018-26671-3)
Supplement: Supplementary file 1 — Supplementary [file 41598_2018_26671_MOESM1_ESM.pdf]

Amblyopia, Strabismus and Refractive Errors in Congenital Ptosis: a systematic review and meta-analysis

Yijie Wang, Yufeng Xu, Xi Liu, Lixia Lou and Juan Ye*

Supplementary. Table S1. Quality assessment of the included studies.

Study	Define the source of information	List inclusion and exclusion criteria for exposed and unexposed subjects	Indicate time period used for identifying patients	Indicate whether or not subjects were consecutive if not population-based	Indicate if evaluators of subjective components of study were masked to other aspects of the status of the participants	Describe any assessments undertaken for quality assurance	Explain any patient exclusions from analysis	Describe how confounding was assessed and/or controlled	If applicable, explain how missing data were handled in the analysis	Summarize patient response rates and completeness of data collection	Clarify what follow-up, if any, was expected and the percentage of patients for which incomplete data or follow-up was obtained	Quality
Anderson RL[28]	yes	yes	yes	yes	yes	yes	yes	yes	no	no	no	8
Anderson RL[10]	yes	yes	yes	yes	yes	yes	yes	yes	no	no	no	8
Griepentrog GJ[11]	yes	yes	yes	yes	no	yes	yes	yes	no	no	no	7
Griepentrog GJ[29]	yes	yes	yes	yes	no	yes	yes	yes	no	no	no	7
Berry-Brincat A[5]	yes	yes	yes	yes	no	yes	yes	yes	no	no	yes	8
Dray JP[12]	yes	yes	yes	unclear	no	yes	yes	yes	no	no	no	6
Gautam P[13]	yes	yes	yes	unclear	no	yes	no	no	no	no	no	4
Júnior G[30]	yes	yes	yes	unclear	no	yes	no	no	no	no	no	4
Harrad RA[14]	yes	yes	yes	yes	no	no	no	no	no	no	no	4
Hornblass A[15]	yes	yes	yes	no	yes	yes	yes	yes	no	no	no	7
Ho YF[31]	yes	yes	yes	unclear	yes	yes	yes	yes	no	no	no	7
Merriam WW[16]	yes	yes	yes	no	no	yes	yes	yes	no	yes	yes	8
Skaat A[17]	yes	yes	yes	no	no	yes	yes	yes	no	no	no	6
Srinagesh V[18]	yes	yes	yes	unclear	no	yes	yes	yes	no	no	yes	7
Handor H[19]	yes	yes	yes	unclear	no	no	yes	yes	no	no	no	6
Huo L[20]	yes	yes	yes	unclear	yes	yes	yes	yes	no	no	no	7
Abolfazl K[21]	yes	yes	yes	unclear	yes	yes	yes	yes	no	no	no	7
Lin LK[22]	yes	yes	yes	unclear	no	yes	yes	no	no	no	yes	6
Stark N[23]	yes	yes	yes	unclear	no	yes	yes	no	no	no	no	5
Stein A[24]	yes	yes	yes	unclear	yes	yes	yes	yes	no	no	no	7
Thapa R[25]	yes	yes	yes	yes	yes	yes	yes	yes	no	no	no	8
Hashemi H[26]	yes	yes	yes	yes	yes	yes	yes	yes	no	yes	no	9
Rong H[27]	yes	yes	yes	yes	yes	yes	yes	yes	no	no	no	8
Yalaz M[32]	yes	yes	yes	unclear	no	no	yes	no	no	no	no	4

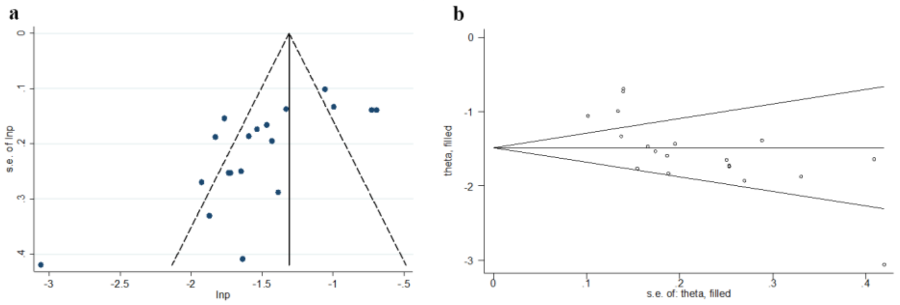

Supplementary. Figure S1. Publication bias testing for amblyopia in congenital ptosis. (a) Funnel plots. (b) Funnel plots using the trim and fill method.

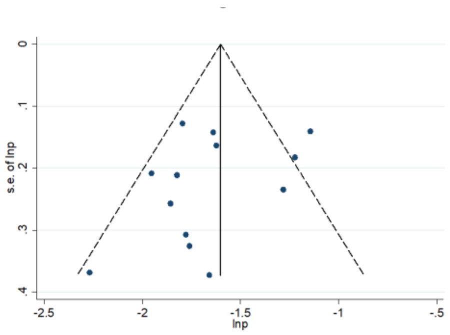

Supplementary. Figure S2. Publication bias testing for strabismus in congenital ptosis, funnel plots.

Supplementary. Appendix A. Search Strategy

PubMed Search Strategy

1. ((((((ptosis*[Title/Abstract]) OR ptoses*[Title/Abstract]) OR blepharoptoses*[Title/Abstract]) OR droopy eyelid*[Title/Abstract]) OR drooping eyelid*[Title/Abstract])) OR "Blepharoptosis"[Mesh])
2. (("Amblyopia"[Mesh]) OR ((Lazy Eye[Title/Abstract]) OR weak sight[Title/Abstract])))
3. ((((((((((((((strabism[Title/Abstract]) OR Squint[Title/Abstract]) OR heterotropia[Title/Abstract]) OR Phorias[Title/Abstract]) OR Phoria[Title/Abstract]) OR Hypertropia[Title/Abstract]) OR Hypertropias[Title/Abstract]) OR Esotropia*[Title/Abstract]) OR Esodeviation*[Title/Abstract]) OR Cross-Eye[Title/Abstract]) OR Esophoria[Title/Abstract]) OR Exotropia[Title/Abstract]) OR Exodeviation*[Title/Abstract]) OR Exophoria[Title/Abstract])) OR "Strabismus"[Mesh])))
4. ((((((((((ametropia[Title/Abstract]) OR Astigmatism[Title/Abstract]) OR Myopia*[Title/Abstract]) OR Nearsightedness*[Title/Abstract]) OR Hyperopia[Title/Abstract])) OR "Refractive Errors"[Mesh]))

Final PubMed search
(1) AND ((2 OR 3) OR 4)
